# Reliability of remote at-home oscillometric blood pressure monitoring in community-dwelling children aged 3–17

**DOI:** 10.3389/fped.2025.1565266

**Published:** 2025-06-04

**Authors:** Emily H. Ho, Berivan Ece, Caroline Clingan, Anne Zola, Zutima Tuladhar, Magdalena Ewa Kupczyk, Linda S. Adair, Richard Gershon

**Affiliations:** ^1^ Department of Medical Social Sciences, Feinberg School of Medicine, Northwestern University, Chicago, IL, United States; ^2^ Department of Nutrition, Gillings Schools of Global Public Health, University of North Carolina, Chapel Hill, NC, United States

**Keywords:** BP measurement, remote measurement, mean arterial pressure, pediatric population, American Academy of Pediatrics (AAP)

## Abstract

**Background:**

As hypertension becomes more prevalent, remote assessment of blood pressure (BP) has been proposed as a method to improve BP management in the pediatric population. We investigated the reliability of at-home BP monitoring in children ages 3–17.

**Methods:**

This study was conducted at six sites across the United States. Children participated in three BP measurements on one occasion by caregivers at home and, on another separate occasion, by trained examiners in a clinic setting. The results were averaged and classified according to the 2017 Pediatric Hypertension Guidelines as normal BP, elevated BP, stage 1 hypertension, or stage 2 hypertension. We collapsed participants with elevated BP, stage 1 hypertension, or stage 2 hypertension into one group: above-normal. We examined the agreement between the caregivers’ and examiners’ BP readings and the ease of the measurement process.

**Results:**

One hundred eighteen (118) children participated in this study (48.3% male; mean age 9.65 ± 4.52 years). Most caregivers (78%−93%) and examiners (88%−99%) rated elements of BP measurement as “easy” or “very easy”. Caregiver and examiners’ agreement on BP classification as normal or above-normal ranged from 75.00% to 90.16% across age groups. Caregiver and examiner BP concordance significantly differed by age group (*p* = .03) and was lower among children with above-normal BPs.

**Conclusions:**

Overall, most aspects of the remote BP measurement process were rated as easy, suggesting that remote monitoring of BP in children is feasible. Concordance of BP measurements by caregivers and examiners was high for children in the normal BP range. More research is needed on the reliability of home BP monitoring across the pediatric age range for those with above-normal BP.

## Introduction

Hypertension is an important risk factor for cardiovascular disease (CVD), a leading cause of death in the United States (US) (1). As of 2020, hypertension affects 48.1% of US adults (2). Initially considered an adult condition, primary hypertension now increasingly impacts children. Studies estimate the prevalence of pediatric hypertension to be between 2% and 6% (3), with primary hypertension now accounting for most cases of pediatric hypertension (4–6). Early tracking of BP during childhood is important because childhood hypertension can predict hypertension in adulthood (7, 8), as well as adult CVD and mortality (9–11). Thus, tracking and controlling high BP in childhood and adolescence may mitigate and prevent adult CVD.

Though early tracking and diagnosis are important, implementation poses challenges. Pediatric BP measurement in clinics is impacted by the white coat effect, where children display higher BPs in clinic than at home (9, 10, 12), as well as masked hypertension (13), where BPs are within normal ranges in clinic but elevated at home (12). To mitigate these two biases, Clinical Practice Guidelines from the American Academy of Pediatrics (AAP) recommend obtaining BP measurements on three separate occasions to diagnose hypertension. However, pragmatically, this is difficult – in a study of obese pediatric patients, nearly 40% of the sample did not return for a follow-up BP visit within 1–2 weeks (14), suggesting repeated clinic visits are not feasible.

To address the logistical hurdles of frequent in-person BP assessments, there has been recent interest in monitoring BP at home. Home BP monitoring may be a less stressful, cost effective (15–17), more natural environment for children, potentially yielding more accurate estimates. It allows for BP measurements over several days without clinic visits, providing a more diagnostically complete picture of hypertension while being highly cost effective (15). Additionally, home BP monitoring is less costly, more accessible, and more acceptable by patients compared with ambulatory methods, which require devices that may limit daily activities and sleep (18, 19).

Despite the potential advantages, evidence on the concordance of home BP measurements with clinic measurements in pediatric populations is limited. In the present study, concordance refers to the agreement between two or more measurements. A recent study comparing home BPs with clinic BPs was limited by significant loss to follow-up, with only 26 of 72 participants completing clinic BP measurements and submitting home BP measurements; concordance between home and clinic BPs was low (50%) within the sample of 26 (10). In another study of 102 children aged 6–18, home BP had high concordance with ambulatory BP monitoring (80%) and was a useful test for detecting white-coat hypertension (20). Studies to date have been limited by small sample sizes, attrition, and limited age ranges. More research is needed on the feasibility and reliability of home BP monitoring in pediatric patients, especially given the increasing prevalence of pediatric hypertension and the increasing use of telehealth technologies (10, 16).

### The current study

This study is part of a larger multisite study (21) evaluating the reliability of anthropometric measurements (e.g., height and weight) taken remotely at home compared with those taken at a study site. In this study, our objective is to evaluate the feasibility and concordance of BP measurements taken by caregivers at home compared with BP measurements taken by trained examiners at a study site.

## Methods

This study protocol was approved by WIRB-Copernicus Group (WCG; #20231258). Before beginning study enrollment, a team of English-Spanish bilingual speakers translated the study materials through two independent forward translations (English to Spanish) and one independent back translation (Spanish to English), followed by reconciliation, review, and proofreading. Participants were recruited through a market panel company with specific age, gender, race/education, and mother education targets (21) across six study sites across the United States. Participants underwent an informed consent process, and then they were randomly assigned to have their measurements completed first at their study site or at home (50% were randomly selected to complete the study site assessment by examiner first, the other 50% at home by caregiver). Study site measurements were completed by examiners. The examiners were research staff hired from a market panel research company (SAGO), and were trained and certified by a pediatric nurse to collect blood pressure measurements in children. Data collection was completed in six SAGO study facilities across the US. All six facilities had dedicated private multi-purpose rooms with blood pressure equipment (i.e., a full set of cuffs of various sizes blood pressure monitor, tape for measuring arm circumference, alcohol wipes, etc.) and appropriate furniture (e.g., chair, table). Home measurements were completed by family caregivers, who received a comprehensive step-by-step instructional manual (see Supplementary 2) and other resource documents.

Before BP measurements, children underwent a five-minute rest period with a book or online video. Children were instructed to have their feet flat on the floor or a step stool. Administrators were instructed to use the right arm. The time of day for BP measurement was not prescribed, though it was recorded on the data collection forms. Mid-arm circumference was measured twice, and the mean was used to determine the appropriate cuff size. Caregivers and examiners measured BP three times with a one-minute rest between them. They also measured height three times and weight two to three times, depending on how great the discrepancy between the first two measurements was, as outlined in the instructional manual (see Supplementary 2).

After completing all measurements, administrators answered survey questions on the ease of use of the BP measurement process on a five-point Likert scale and provided open-ended feedback. Measurements and survey responses were recorded on paper data collection forms and entered into an online system twice for consistency. Random checks ensured that paper-pencil and online entries matched.

To calculate body mass index (BMI), all measurements across caregivers and examiners were averaged for height and weight. We report the high reliability of weight and height between the two modes of administration in another paper (Ho et al., provisionally accepted). We used R version 4.3.1 and the *cdcanthro* package (22) to calculate BMI percentiles based on age- and sex-specific norms. BMI percentiles were used to classify whether the participant was underweight, normal weight, overweight, and obese according to AAP guidelines on the evaluation and management of pediatric obesity (23).

### Equipment

The 2000-A Welch Allyn ProBP 2000 Digital Blood Pressure Device was chosen for its ease of use, portability, and validation for in-person use for adults and children (24). As accurate cuff size is essential for accurate BP readings (25), multiple cuff sizes were provided to ensure accuracy, especially for younger children.

### Statistical analysis

BPs were classified per the AAP's 2017 Clinical Practice Guideline on pediatric hypertension using the associated MDCalc tool “AAP Pediatric Hypertension Guidelines” (26, 27). For children ages 3–12, the BP classifications are defined as follows: normal BP [systolic blood pressure (SBP) and diastolic blood pressure (DBP) <90th percentile], elevated BP (SBP or DBP ≥90th percentile and <95th percentile), stage 1 hypertension (SBP or DBP ≥95th percentile and <95th percentile plus 11mmHg), stage 2 hypertension (SBP or DBP ≥95th percentile plus 12 mmHg). For children ages 13–17, the BP classifications are defined as follows to align with adult definitions of hypertension: normal BP (<120/<80mmHg), elevated BP (≥120/<80–129/<80 mmHg), stage 1 hypertension (130–139/80–89mmHg), stage 2 hypertension (≥140/≥90 mmHg) (27).

In accordance with AAP guidelines, if the first BP measurement was a normal BP, then the participant was classified as having normal BP; if the first BP measurement was an abnormal BP (i.e., in the elevated or hypertensive range as previously defined), it was discarded, and the second and third BPs were averaged and used for BP classification (27). BP classification was completed twice: once with caregiver-measured BPs and once with examiner-measured BPs. Four participants had first BP measurements that were out-of-range for the MDCalc tool; these measurements were discarded, and the second and third measurements were averaged for these patients to guide their BP classifications.

We used SBP and DBP to calculate mean arterial pressure (MAP), the average vascular resistance throughout one cardiac cycle, defined as MAP = DBP + (⅓)(SBP-DBP) (28). We calculated and used MAP as we preferred having one measure that combines SBP and DBP. If the first BP was normal, the corresponding MAP was used; if abnormal, MAPs from the second and third BPs were averaged. Each participant had both a caregiver-measured and an examiner-measured MAP. Bland-Altman plots were used to examine the limits of agreement (LOA) for SBP, DBP, and MAP between caregiver and examiner measurements. We conducted two Chi-squared tests to determine whether the time of measurement – AM or PM – was significantly associated with the BP classification or cuff concordance between the administration modes.

We calculated sensitivity, specificity, and percent agreement to examine the inter-rater agreement of BP classifications, using the trained in-person staff as the gold standard. Even though clinic BP measurements can be influenced by the white coat effect or by masked hypertension, we used the on-site measurements by examiners as the gold standard in this study because clinic measurements are often used clinically as the gold standard in pediatrics and clinic measurements are the next best measurement method after 24-hour ambulatory BP monitoring, which is outside the scope of this study. Due to small sample sizes in elevated BP, stage 1 hypertension, and stage 2 hypertension categories, we collapsed the groups as follows: normal BP is “normal”, and elevated BP, stage 1 hypertension, and stage 2 hypertension are “above-normal”. Because of the distinction between children and adolescents (ages 13 and above) in the AAP guidelines, and because toddlers clinically differ from children in many ways, we divided our participants into the following age groups for these analyses: 3–4 years, 5–12 years, and 13–17 years. To examine whether caregiver-examiner agreement varied by covariates (i.e., age group, sex, BMI, and language), we used Fisher's exact test with a Monte Carlo simulation of 2,000 replications.

We calculated descriptive statistics for ease-of-use questions and compared the proportion of caregivers and examiners who endorsed each of the five categories (from “1 = very easy” to“5 = very hard”). We also examined the relationship between caregivers’ reported ease of use and differences between caregiver and examiner BP measurements using Bland-Altman plots.

## Results

Across six study sites, 118 children ages 3 and up (M = 9.65, SD = 4.52; 51.7% female) completed the assessments. Self-reported race and ethnicity yielded a sample of 58.5% White or Caucasian, 31.2% Black or African American, and 32.2% Hispanic. Spanish data collection forms were completed by 26.3% (*N* = 31) of the sample. Most caregivers in this study were biological mothers of the enrolled children (*N* = 94; 79.7%) (see Table 1). An average of 3.28 days (SD = 3.39) elapsed between measurements at home and those done at a study site.

**Table 1:** Demographic characteristics by age group.

	3–4 years (*N* = 24)	5–12 years (*N* = 61)	13–17 years (*N* = 33)	Overall (*N* = 118)
Site				
Orlando	0 (0%)	5 (8.2%)	3 (9.1%)	8 (6.8%)
Atlanta	5 (20.8%)	17 (27.9%)	5 (15.2%)	27 (22.9%)
Dallas	9 (37.5%)	10 (16.4%)	7 (21.2%)	26 (22.0%)
Houston	5 (20.8%)	12 (19.7%)	5 (15.2%)	22 (18.6%)
Baltimore	0 (0%)	8 (13.1%)	3 (9.1%)	11 (9.3%)
Nashville	5 (20.8%)	9 (14.8%)	10 (30.3%)	24 (20.3%)
Child sex				
Male	15 (62.5%)	26 (42.6%)	16 (48.5%)	57 (48.3%)
Female	9 (37.5%)	35 (57.4%)	17 (51.5%)	61 (51.7%)
Child age (years)				
Mean (SD)	4.06 (0.572)	8.64 (2.37)	15.6 (1.42)	9.65 (4.52)
Median [min, max]	3.89 [3.09, 5.00]	8.65 [5.05, 12.9]	15.8 [13.0, 17.7]	9.22 [3.09, 17.7]
Race				
White or Caucasian	13 (54.2%)	37 (60.7%)	19 (57.6%)	69 (58.5%)
Black or African	6 (25.0%)	17 (27.9%)	11 (33.3%)	34 (28.8%)
American	1 (4.2%)	0 (0.0%)	1 (3.0%)	2 (1.7%)
Asian	1 (4.2%)	0 (0.0%)	0 (0.0%)	1 (0.9%)
American Indian or Native Alaskan				
Other	1 (4.2%)	1 (1.6%)	1 (3.0%)	3 (2.5%)
More than one	2 (8.3%)	6 (9.8%)	1 (3.0%)	9 (7.6%)
Ethnicity				
Not Hispanic	13 (54.2%)	43 (70.5%)	24 (72.7%)	80 (67.8%)
Hispanic	11 (45.8%)	18 (29.5%)	9 (27.3%)	38 (32.2%)
Language				
English	18 (75.0%)	44 (72.1%)	25 (75.8%)	87 (73.7%)
Spanish	6 (25.0%)	17 (27.9%)	8 (24.2%)	31 (26.3%)
Child BMI				
Underweight	2 (8.3%)	1 (1.6%)	2 (6.1%)	5 (4.2%)
Normal weight	15 (62.5%)	37 (60.7%)	13 (39.4%)	65 (55.1%)
Overweight	5 (20.8%)	14 (23.0%)	10 (30.3%)	29 (24.6%)
Obese	2 (8.3%)	9 (14.8%)	8 (24.2%)	19 (16.1%)
Caregiver BP class				
Normal	14 (58.3%)	58 (95.1%)	29 (87.9%)	101 (85.6%)
Elevated	2 (8.3%)	2 (3.3%)	2 (6.1%)	6 (5.1%)
Stage 1	7 (29.2%)	1 (1.6%)	1 (3.0%)	9 (7.6%)
Stage 2	1 (4.2%)	0 (0%)	1 (3.0%)	2 (1.7%)
Examiner BP class				
Normal	14 (58.3%)	54 (88.5%)	29 (87.9%)	97 (82.2%)
Elevated	3 (12.5%)	4 (6.6%)	2 (6.1%)	9 (7.6%)
Stage 1	5 (20.8%)	3 (4.9%)	2 (6.1%)	10 (8.5%)
Stage 2	2 (8.3%)	0 (0%)	0 (0%)	2 (1.7%)
Caregiver type				
Biological mother	16 (66.7%)	52 (85.2%)	26 (78.8%)	94 (79.7%)
Biological father	6 (25.0%)	8 (13.1%)	1 (3.0%)	15 (12.7%)
Other	2 (8.3%)	1 (1.6%)	5 (15.2%)	8 (6.8%)
Missing	0 (0%)	0 (0%)	1 (3.0%)	1 (0.8%)
Caregiver age				
Mean (SD)	37.2 (6.99)	40.2 (7.02)	44.1 (7.35)	40.7 (7.42)
Median [min, max]	39.0 [22.0, 50.0]	41.0 [21.0, 53.0]	43.0 [34.0, 66.0]	41.0 [21.0, 66.0]
Missing	3 (12.5%)	2 (3.3%)	4 (12.1%)	9 (7.6%)
Caregiver education				
< High school graduate	3 (12.5%)	4 (6.6%)	6 (18.2%)	13 (11%)
High school graduate/GED	4 (16.7%)	8 (13.1%)	4 (12.1%)	16 (13.6%)
Some college	7 (29.2%)	10 (16.4%)	5 (15.2%)	22 (18.6%)
2 years college graduate	0 (0.0%)	5 (8.2%)	5 (15.2%)	10 (8.5%)
College graduate	5 (20.8%)	25 (41.0%)	8 (24.2%)	38 (32.2%)
Graduate degree	4 (16.7%)	9 (14.8%)	5 (15.2%)	18 (15.3%)
Missingness	1 (4.2%)	0 (0.0%)	0 (0.0%)	1 (0.8%)

We first analyzed BMI and BP classifications for all participants. In nearly all cases, the mean absolute deviations (MAD) in height and weight were small and similar across the age ranges (height: MAD = 1.37, mean SD = 1.56; weight: MAD = 0.52, mean SD = 0.60), making height and weight reliable variables for intermediary inputs to BMI calculations. In this study, most children are classified as normal weight (*N* = 65; 55.1%), followed by overweight (*N* = 29; 24.6%), obese (*N* = 19; 16.1%), and underweight (*N* = 5; 4.2%). The majority of the sample exhibited normal BP according to both caregivers (*N* = 101; 85.59%) and examiners (*N* = 97; 82.20%). Caregivers and examiners measured elevated BPs in 5.08% (*N* = 6) and 7.63% (*N* = 9) and BPs consistent with stage 1 hypertension in 7.63% (*N* = 9) and 8.47% (*N* = 10) of children, respectively. Caregivers and examiners alike reported 2 (1.69%) cases of BPs consistent with stage 2 hypertension.

The proportion of examiners and caregivers who chose the same cuff size was high at 73.68%. An additional 23.69% deviated by only one cuff size: 15.79% of caregivers selected a larger size than the examiners, while 10.53% of examiners selected a larger size than the caregivers. There was no significant relationship between cuff size agreement/disagreement and the mean absolute difference between caregiver and examiner by the following indices: (1) SBP [*t*(112) = 1.08, *p* = .28, Cohen's *d* = −0.23, 2] DBP [*t*(112) = 0.51, *p* = .61, Cohen's *d* = −0.11], or 3) MAPs [*t*(112) = 0.89, *p* = .38, Cohen's *d* = −0.19].

**Figure 1:**
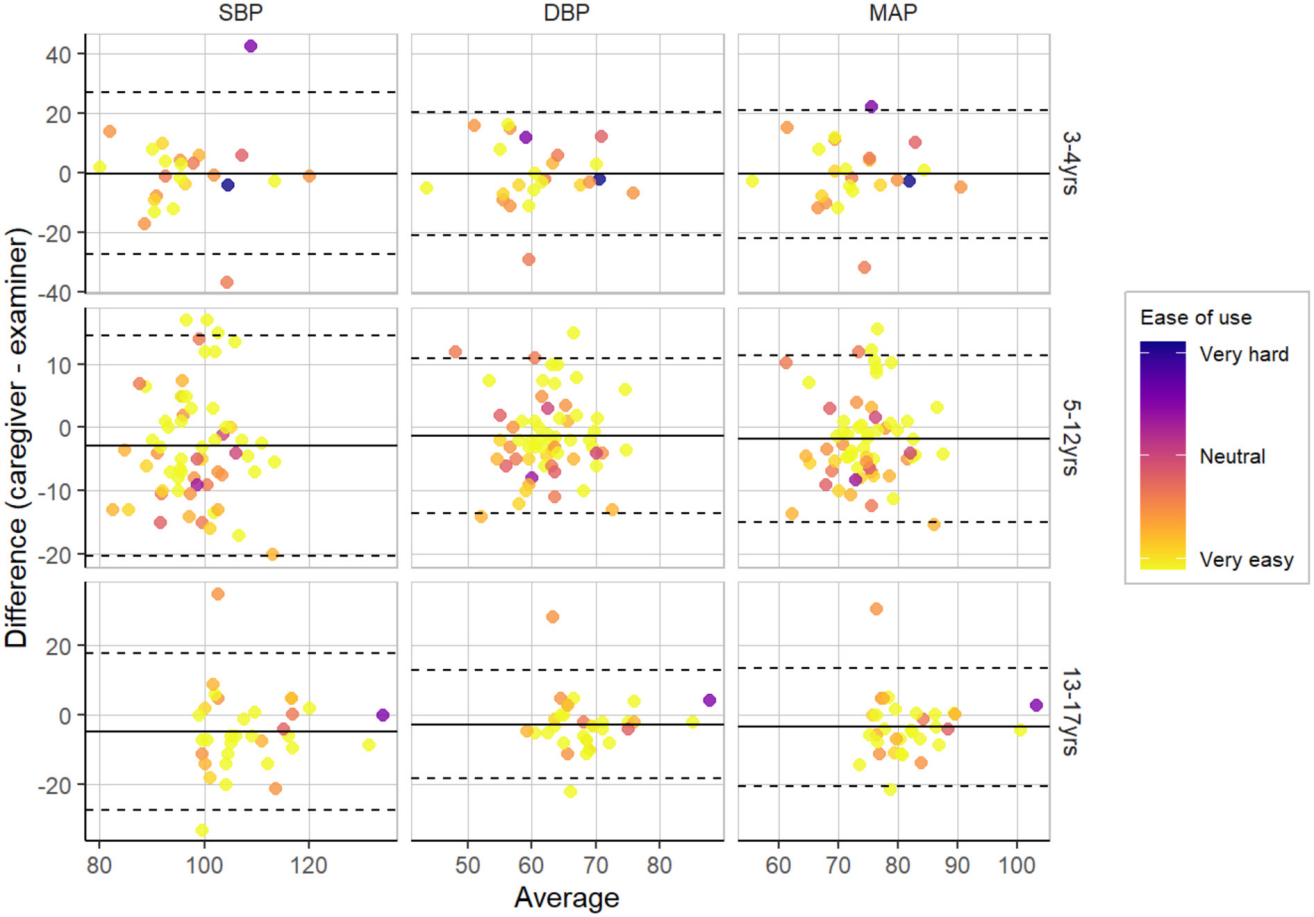
Bland-Altman plots for BP concordance by caregiver ease of use across three age groups, with LOAs from ± 1.96 SD.

We examined concordance between average BPs as measured by caregivers and examiners. We found moderate correlations between SBPs (*r* = .49), DBPs (*r* = .50), and MAPs (*r* = .51) derived from caregiver and examiner measurements, suggesting moderate concordance and convergent validity in measurement across the two modes. Figure 1 shows Bland-Altman plots for BP concordance by caregiver ease of use across three age groups, with LOAs from ± 1.96 SD. For color coding by different classifications (e.g., normal vs. above-normal BP classifications), refer to Supplementary 1 (Supplementary Figures S1, S2).

We examined the agreement of caregiver and examiner BP classifications as normal or above-normal, where above-normal refers to the collapsed group of elevated BP, stage 1 hypertension, and stage 2 hypertension. We stratified this analysis by the following age groups: 3–4, 5–12, and 13–17 years (Table 2). See Supplementary Materials for analyses by all BP classifications (i.e., using all four classifications - normal BP, elevated BP, stage 1 hypertension, and stage 2 hypertension). Caregiver and examiners’ agreement ranged from 75.00% [95% confidence interval (CI) = 57.68%, 92.32%] to 90.16% (95% CI = 82.69, 97.63%) across age groups. Specificity, or agreement between caregivers and examiners on classifications of normal BP, was high, ranging from 78.57% (95% CI = 57.08%, 100.06%) to 98.15% (95% CI = 94.56%, 101.74%). Fisher's exact test confirmed caregiver and examiner agreement significantly differed by age group, *p* = .03. They exhibited lower agreement among BPs classified as above-normal, especially for older children, with sensitivity values of 28.57% (95% CI = −0.49%, 62.04%) for 5–12 years old and 50.00% (95% CI = 1.00%, 99.00%) for 13–17 years old. In contrast, sensitivity among 3–4-year-old children was acceptable at 70.00% (95% CI = 41.60%, 98.40%). Fisher's exact tests also confirmed the caregiver and examiner agreement did not differ by child's BMI category (*p* = .36), child sex (*p* = .81), participant language (*p* > .99), or randomization condition (*p* = .62).

**Table 2:** Caregiver and examiner concordance between normal and above-normal BP classifications by child age group.

Age (years)	Caregiver	Examiner		Specificity	Sensitivity	Concordance
		Normal	Above-normal			
3–4	Normal	11	3	78.57%	70.00%	75.00%
	Above-normal	3	7			
5–12	Normal	53	5	98.15%	28.57%	90.16%
	Above-normal	1	2			
13–17	Normal	27	2	93.10%	50.00%	87.88%
	Above-normal	2	2			

To examine whether caregiver or examiner measurements were more variable, we calculated within-child standard deviations among SBP and DBP measurements. Using Spearman-rank correlations, the variability of SBPs was moderately correlated with the variability of DBPs for caregivers, *r*(105) = .48, *p* < .001, and for examiners, *r*(101) = .42, *p* < .001, suggesting similar levels of variability regardless of mode of administration. However, caregiver and examiner variabilities were not associated for DBPs, *r*(105) = .12, *p* *=* .22, or SBPs, *r*(98) = .11, *p* *=* .29. There was also no significant effect of the time of measurement on concordance between the two modes on blood pressure classifications [x^2^(1) = 0.045, *p* > .05] nor cuff size measurements [x^2^(1) = 3.30, *p* > .05].

### Ease-of-use questions

**Figure 2:**
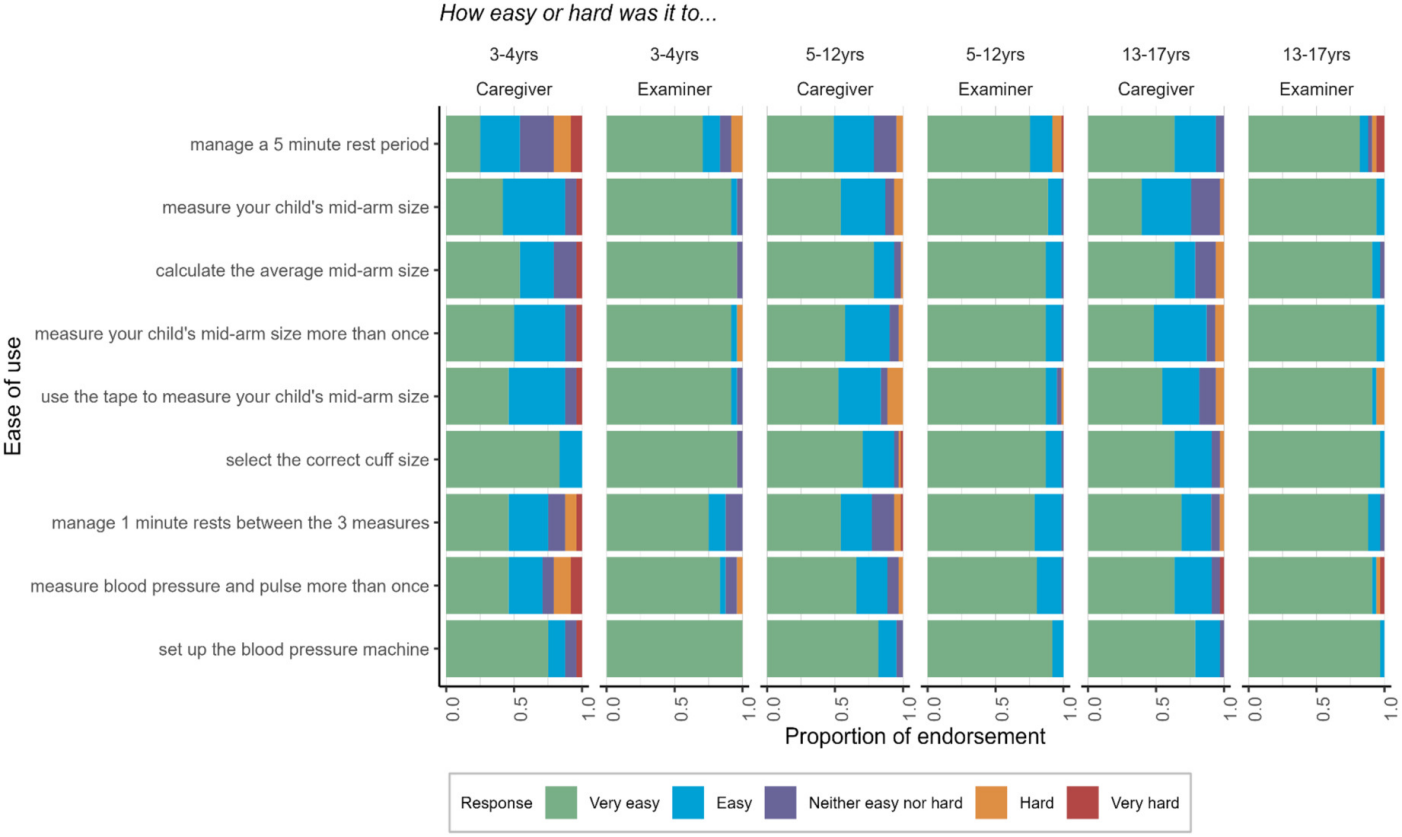
Ease of use of the BP measurement process self-reported by caregivers and examiners across three age groups.

Analysis of the ease-of-use questions suggested that it was not difficult for caregivers/examiners to understand the instructions for conducting the BP measurements and data entry. Figure 2 displays how the proportion of caregivers/examiners rated the ease of use of the BP measurement process. Most caregivers (78%–93%) and examiners (88%–99%) rated the BP measurement process as “very easy” or “easy”. Compared to examiners, caregivers were more likely to report certain tasks as “hard” or “very hard”, although still infrequently (less than 10% of responses). Ease-of-use ratings were similar across age groups except for managing a 5-minute rest period and measuring BP more than once, which were rated as more difficult by caregivers and examiners for children 3–4 years old compared to older ages. We used Fisher's exact tests to examine the BP classification agreement by those who endorsed any survey questions as “hard” or “very hard” compared to those who did not endorse any aspect of administration as hard/very hard. Agreement was higher among caregivers who did not endorse difficulty (86.2%) compared to caregivers who did endorse difficulty (66.7%), *p* = .04. Agreement was similar for examiners who did (72.7%) and did not endorse difficulty (83.2%), *p* = .41, likely due to the examiners’ familiarity with the procedure after repeated assessments across many children. In contrast, caregivers only completed the assessment for one child.

## Discussion

In support of prior work conducted to review the reliability and benefits of remote BP measurement (29, 30), this study shows that at-home BP measurements by caregivers are generally highly concordant with those taken by trained examiners, regardless of covariates, such as the child's sex, BMI, or randomization arm. Furthermore, most caregivers rated the BP measurement process as easy, supporting the feasibility and acceptability of home BP monitoring in pediatrics research (31).

Across age groups, caregivers and examiners agreed more when classifying a child's BP as normal. Agreement on identifying above-normal BP was lower and varied significantly by age group, possibly due to the small number of children with high BPs, especially among those aged 5–17, where only 14 (15%) were identified by either caregiver or examiners. Sensitivity was highest (70.00%) in the 3–4-year-old group, where above-normal BP prevalence was also highest (41.7%). One hypothesis for this difference is that younger children may have had more clearly elevated readings, making abnormal BP easier to detect; in contrast, older children may have had values closer to diagnostic thresholds, contributing to lower sensitivity. Notably, when caregivers and examiners disagreed, there was no clear pattern as to who detected above-normal BP (see Table 2). Overall, these age-related differences should be interpreted cautiously given the small number of abnormal cases. Additional studies with larger samples of children with above-normal BPs are needed to investigate age, along with degree of degree of BP elevation from the diagnostic threshold, as a mediator of the reliability of home BP monitoring.

We found moderate correlations between SBPs (*r* = .49), DBPs (*r* = .50), and MAPs (*r* = .51) derived from caregiver and examiner measurements. Clinically, this means there is some variability in the exact BP measurements obtained by caregivers and examiners, which could affect BP classification at the individual level. However, despite the moderate variability, caregiver and examiner measurements generally agree on the clinical classification of BP. Further standardization or training or repeating BP measurement on different occasions may enhance the accuracy of caregiver assessments, ensuring consistent identification of hypertension for timely intervention.

Hypertensive BP prevalence in this sample was 9%–10%, and above-normal (elevated or hypertensive) BP prevalence in this sample was 14%–18%. While our estimate for above-normal BP aligns with existing literature, our estimate for hypertensive BP prevalence exceeds most estimates (26%) (3, 6, 32–34). This might result from our small sample size or the single BP measurement instance by caregivers and examiners. However, caregivers and examiners detected similar rates of above-normal BP, implying that these rates are not solely due to the measurement method (3). Other factors, such as difficulties in securing required rest periods and challenges in accurate pediatric BP measurement (e.g., white coat effect and masked hypertension), may also contribute and are difficult to fully evaluate without 24-hour ambulatory BP monitoring as a true gold standard comparator. Finally, our sample size had relatively large proportions of Black and Hispanic participants, groups that may experience social risk factors influencing blood pressure. While this was beyond the scope of our study, it warrants consideration in future research.

Overall, caregivers evaluated remote BP monitoring as easy with proper instructions. Less than 10% of caregivers rated any aspect of the BP measurement process as “hard” or “very hard”. Additionally, this study saw no loss to follow-up. In other words, all children who participated in on-site BP measurement also participated in home BP measurement by caregivers and submitted a complete BP dataset. This highlights the feasibility and acceptability of home BP monitoring by caregivers.

The most challenging aspects for caregivers and examiners were (1) measuring and calculating mid-arm cuff size, and (2) managing the rests before and between BP measurements. Even so, caregivers and examiners exhibited high reliability in measuring a child's mid-arm cuff size. And of caregivers who did not match selecting the same cuff size as the examiners’, there was no systemic over or under estimation of cuff size selection amongst the caregivers and/or examiners. However, minimizing these difficulties should be prioritized when implementing home BP monitoring in research or clinical settings. For example, clinicians could consider pre-selecting the appropriate cuff size and prescribing that to caregivers to mitigate the perceived difficulties associated with independent cuff selection by caregivers. To mitigate the difficulties of rest periods, clinicians could consider explaining the rationale for rest periods – i.e., rest periods help ensure the accuracy of BP measurements by minimizing the influence of environmental changes on BP. Alternatively, as caregivers and patients become more experienced with taking home BP measurements, for example, over a series of days, this issue may become less pertinent. This is supported by the fact that BP agreement did not significantly differ when examiners rated any aspect of the measurement process as “hard” or “very hard”, suggesting that their experience with taking BPs in many children may have aided them in taking accurate BP measurements despite perceived difficulties with the measurement process.

Caregivers were less likely to report aspects of the BP measurement process as “very easy” in the 3–4 year-old age group. Notably, the prevalence of above-normal BP among 3- and 4-year-olds in this sample was higher than in other age groups, with 29% (*N* = 7) identified by both caregivers and examiners. Both caregivers and examiners reported greater difficulty managing a 5-minute rest period and multiple BP measurements in toddlers, potentially inflating above-normal BP rates. This suggests that toddlers may be a uniquely challenging group to obtain accurate home BP measurements. As pediatric BP studies frequently do not go down to age 3, more research is needed on best practices for BP monitoring and the feasibility of home BP monitoring for this age group.

Our findings suggest that examiners found it easier to administer blood pressure measurements in person compared to caregivers who conducted them remotely. This discrepancy may be attributed to the hands-on training that examiners received, while caregivers relied solely on guidance documents for support during the measurement process.

### Strengths

This study adds to the literature on pediatric home BP measurement in several ways. The current study captures the entire age range recommended by AAP Blood Pressure Clinical Practice Guideline (3–17), unlike previous studies, which typically start at age 5 or above (e.g., 35, 36). The current protocol adheres to the AAP guidelines, including a five-minute rest before the initial BP measurement and a one-minute rest between the three measurements. To minimize demand effects, participants were randomized to either start with remote or in-person assessment, which is relatively unique to our study. Our sample included Spanish speakers in addition to English speakers to reflect the growing Hispanic population throughout the United States.

### Limitations

Given that we covered the entire age range recommended by the AAP Blood Pressure Clinical Practice Guideline, each age band's sample size was relatively small. Especially for the 3–4-year-old group, the sample size was smaller, with further subdivision into normal/above-normal BP categories. While BP measurements were completed three times per participant by caregiver and examiner, they were conducted at a single time point in each setting, which is insufficient for diagnosing hypertension, according to the AAP (27). Additionally, measurements were completed using oscillometric devices, but clinical gold standard assessments should be confirmed with auscultatory BP measurement (27). This study did not include 24-hour ambulatory BP monitoring, which is the current gold standard for diagnosing hypertension in pediatrics. Without ambulatory BP monitoring, we were unable to assess the rates of masked hypertension or white coat hypertension, which may bias our results. The examiners, while trained and certified by a pediatric nurse, were not clinicians. Finally, the average duration of 3.3 days between home and site measurements may introduce potential variability independent of the measurement method.

## Conclusion

Pediatric hypertension is becoming more prevalent but remains underdiagnosed. This highlights the need for novel BP monitoring strategies to mitigate the health impacts of prolonged hypertension. Home BP monitoring is a valuable tool for hypertension diagnosis and management, especially in the era of telemedicine (37, 38). It can reduce the burden of repeated clinic visits and alleviate children's anxiety about doctor visits. It may also be cost-saving, increase patient compliance, and allow earlier intervention to reduce CVD risk in youth and adulthood. We demonstrated the feasibility and partial reliability of home BP monitoring for pediatric patients ages 3–17, suggesting its utility alongside clinic measurements. More research is needed on remote BP monitoring for ages 3–5, implementation strategies, and its effect on diagnosing and managing pediatric hypertension.

## Data Availability

The raw data supporting the conclusions of this article will be made available by the authors, without undue reservation.
