# Supplementary material for: Reliability of remote at-home oscillometric blood pressure monitoring in community-dwelling children aged 3–17
Source: Front Pediatr. 2025 Jun 4;13:1565266. doi: 10.3389/fped.2025.1565266 (PMC12174106; doi:10.3389/fped.2025.1565266)
Supplement: Supplementary file 1 [file Datasheet1.pdf]

SUPPLEMENTAL MATERIAL

S1: Bland-Altman plots by child BMI category

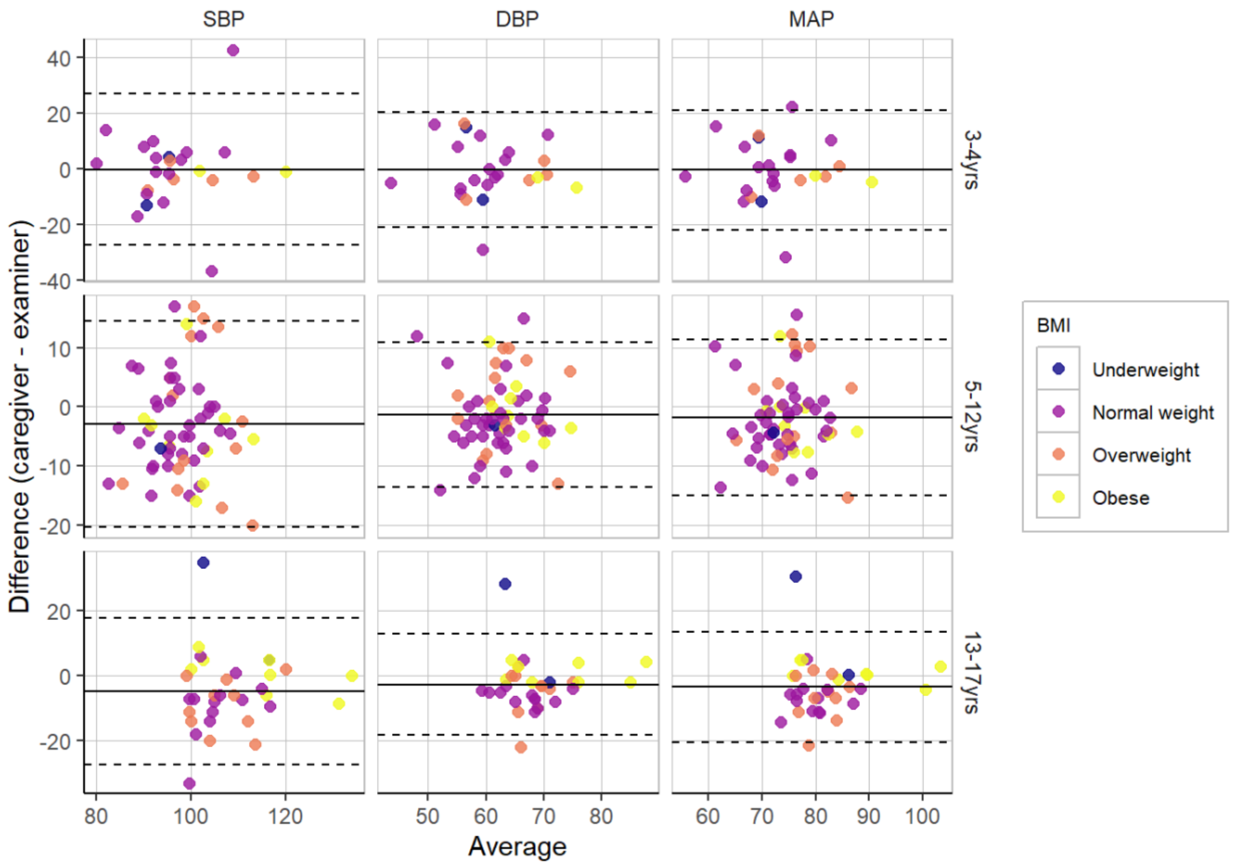

S2: Bland-Altman plots by BP classification

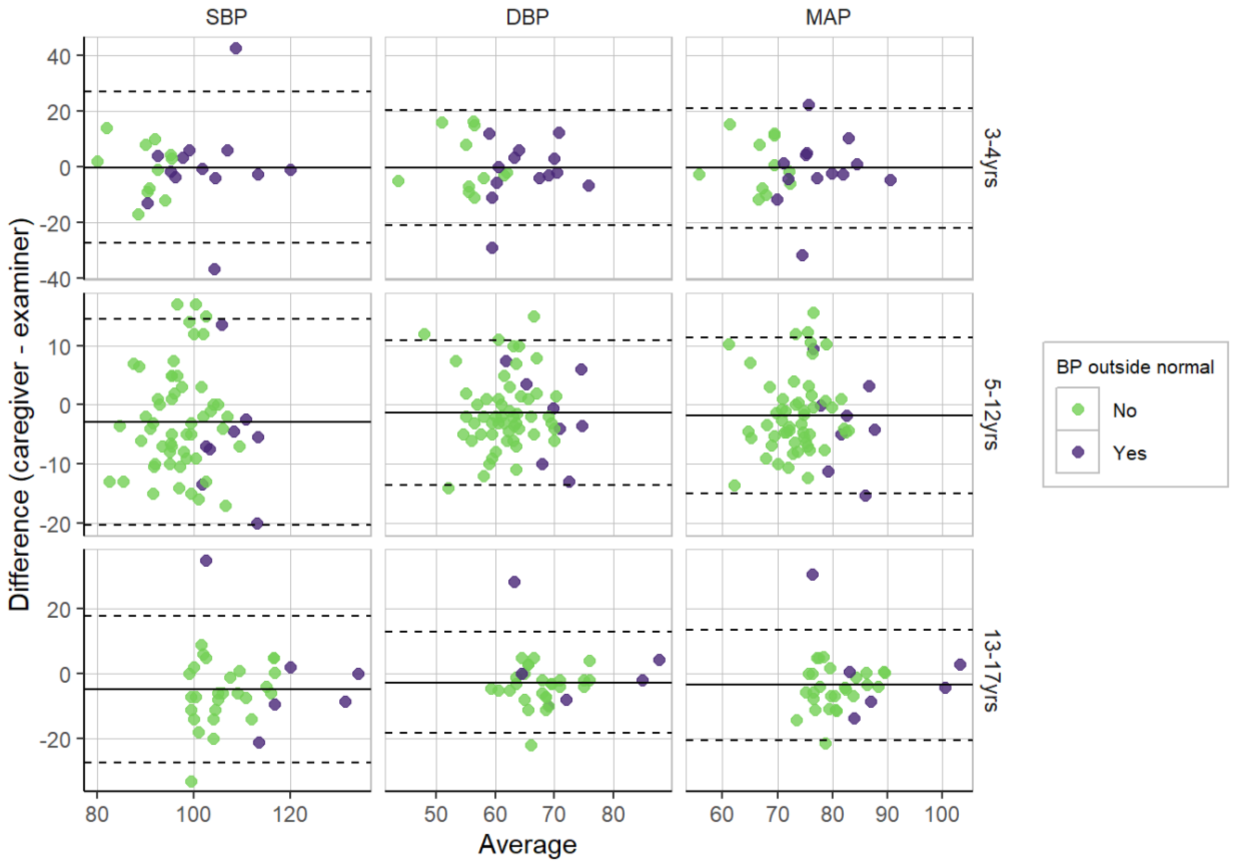

Note: BP is considered outside of normal if either caregiver or examiner classified BP as Elevated, Hypertension Stage 1, or Hypertension Stage 2.

S3: Caregiver and examiner agreement between all BP classifications

		Examiner				Kappa		ICC	
Age (years)	Caregiver	Normal	Elevated	Stage 1	Stage 2	Statistic	p	Statistic	p
3-4	Normal	11	1	1	1	0.36	.033	0.34	.047
	Elevated	0	1	1	0				
	Stage 1	2	1	3	1				
	Stage 2	1	0	0	0				
12-15	Normal	53	2	3	0	0.24	.013	0.22	.040
	Elevated	1	1	0	0				
	Stage 1	0	1	0	0				
	Stage 2	0	0	0	0				
13-17	Normal	27	2	0	0	0.58	<.001	0.78	<.001
	Elevated	2	0	0	0				
	Stage 1	0	0	1	0				
	Stage 2	0	0	1	0				
