# Supplementary material for: Reliability of remote at-home oscillometric blood pressure monitoring in community-dwelling children aged 3–17
Source: Front Pediatr. 2025 Jun 4;13:1565266. doi: 10.3389/fped.2025.1565266 (PMC12174106; doi:10.3389/fped.2025.1565266)
Supplement: Supplementary file 2 [file Datasheet2.pdf]

SUPPLEMENTARY MATERIAL 2

Some images within the document have been redacted to remove identifiable individuals. Please contact corresponding author if there are any questions.

CAREGIVER INSTRUCTIONS FOR BP MEASUREMENT

(A) Blood Pressure

Device: Blood Pressure Machine and Blood Pressure Cuffs
(This measurement requires that your child sit in one place for a longer period. You can have your child watch a familiar video, play with their favorite toy, or watch a sibling to help while measuring your child)

Step 1: Place the blood pressure machine and cuffs on the table or flat surface.

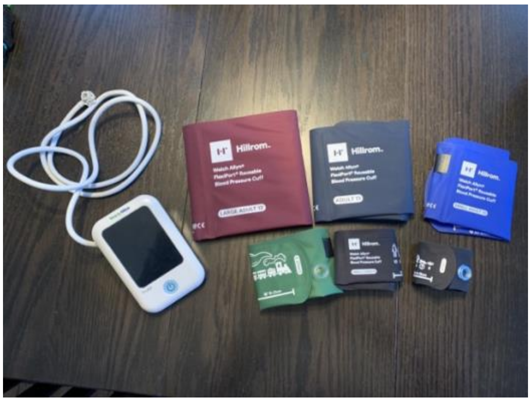

Step 2: Remove any sleeves covering the upper arm. Have the child sit with his/her right arm supported on the table, child's feet should be resting flat on the floor or on a step stool.

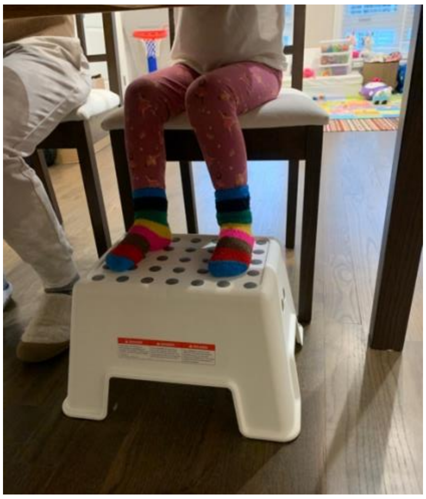

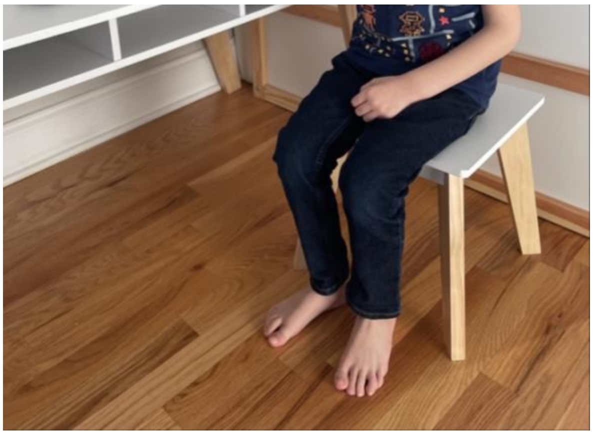

Step 3: Measure the mid-arm size of the child by using the centimeter side of the body measuring tape. The mid-arm can be located between the shoulder and elbow. Wrap the tape measure around the mid-arm to measure.

[redacted]

Step 4: Write down the mid-arm measurement in Data Collection Form (DCF).

Step 5: Repeat the mid-arm measurement for the same arm and write down the measurements in the Data Collection Form (DCF).

Step 6: Calculate the average of the two mid-arm size measurements you measured. For calculating the average, you should add the two measurements and then divide their total by 2:

(a + b) / 2 = Average (please write down the average mid-arm size in the Data Collection Form)

For calculation, use a calculator (e.g., located on your smartphone, iPad etc.)

Step 7: Now compare the number you calculated with the cuff size ranges below.

For example, if your average number came out to 18cm, you would select the '***Size 9 green cuff***' provided in your kit OR if your average number came out to 16cm, you could select either the ***'Size 8 black cuff'*** or the ***'Size 9 green cuff'***.

9-13cm Size 7: Darker Black

12-16cm Size 8: Black

15-21 cm Size 9: Green

20-26 cm Size 10: Blue

25-34 cm Size 11: Gray

32-43 cm Size 12: Red

Make sure you select the correct cuff size for accurate blood pressure measurement. If you are using **Size 7 or Size 8** cuffs, please double check the cuff before use as both **Size 7 and Size 8** cuffs are black in color.

(A) Blood Pressure Continued

Step 8: Mark the appropriate blood pressure cuff size listed in the Data Collection Form (DCF).

Step 9: Place the BP cuff around the arm at the level of the heart. Apply the cuff, allowing room for no more than two fingers. Insert the end of blood pressure connection tube into the cuff as shown below.

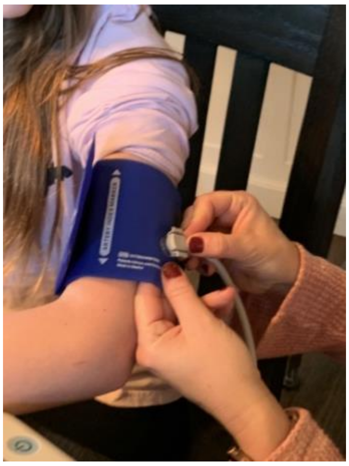

Step 10: Once the cuff is placed, allow the child to sit for five minutes. You can have your child watch a familiar video, play with their favorite toy, or watch a sibling to help while measuring your child.

Step 11: Press the start button on the machine and wait for the machine to display the numbers of the screen.

Step 12: During this time, check the child's positioning. Arm and fingers should be relaxed, palm facing up, cuff at heart level, and make sure the cuff is secure. The measurement will take 20 seconds.

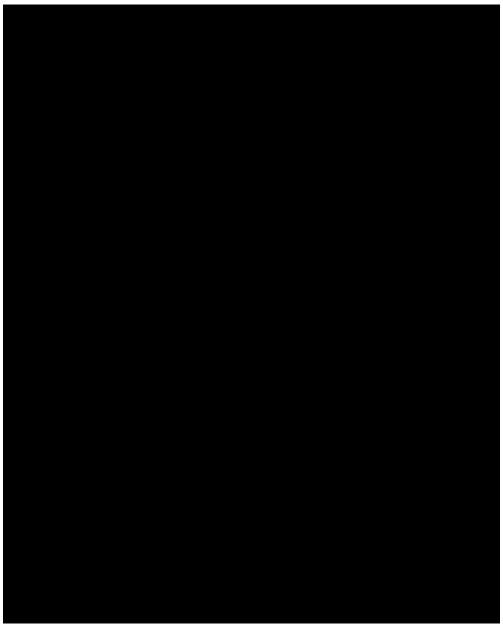

Note: If your child is not sitting still, you will see the sign shown in the picture below.

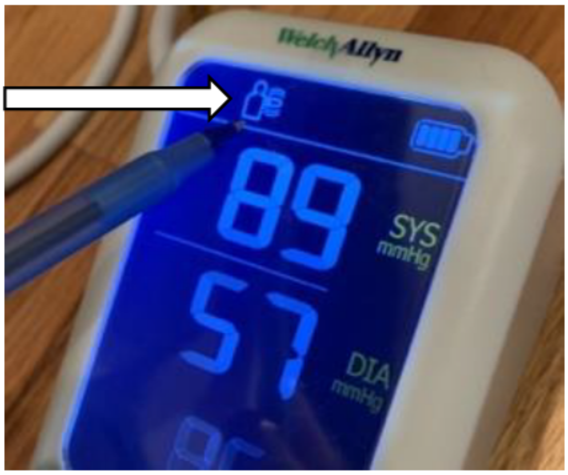

Step 13: Write down the SYS (Systolic BP), the DIA (Diastolic BP), the pulse rate in the instrument in section Data Collection Form (DCF) provided to you. After this step, you can remove the child's cuff.

Step 14: Repeat steps (8-13) two more times in 1-minute intervals between each measurement, and write down a total of three SYS (Systolic BP), the DIA (Diastolic BP), the pulse in the instrument in the boxes with the same name next to it on the Date Collection Form (DCF)

Step 15: After the third measurement is noted in the Data Collection Form, remove the BP cuff from the child's arm and the measurement is complete.

EXAMINER INSTRUCTIONS FOR BP MEASUREMENT

Blood Pressure Measurement (3-17y)

Device: Blood Pressure Machine

(This measurement requires that the child sit in one place for a longer period. You can have the child watch a familiar video, play with their favorite toy, or watch a sibling to help while measuring the child)

Step 1: Place the blood pressure machine and cuffs on the table or flat surface.

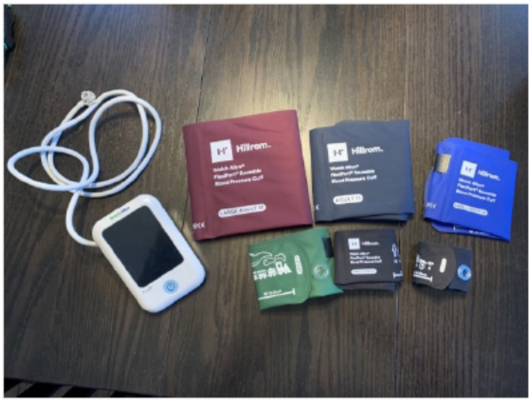

Step 2: Remove any sleeves covering the upper arm. Have the child sit with his/her right arm supported on the table, child's feet should be resting flat on the floor or on a step stool.

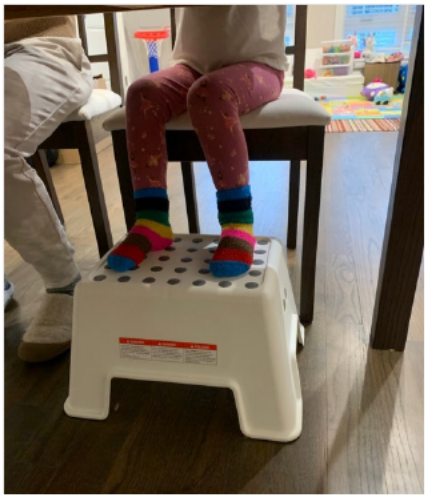

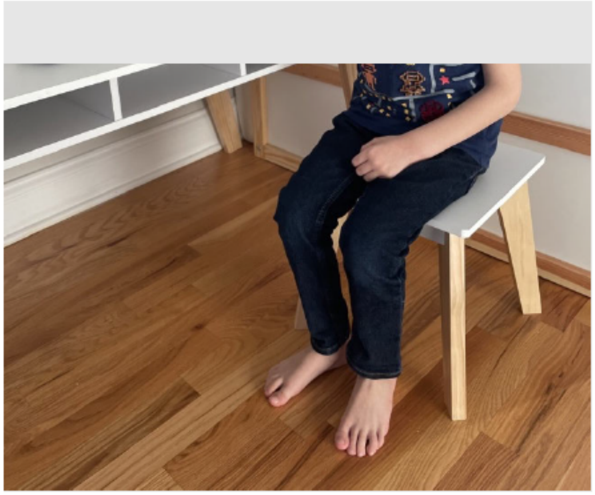

Step 3: Measure the mid-arm size of the child by using the centimeter side of the body measuring tape. The mid-arm can be located between the shoulder and elbow. Wrap the tape measure around the mid-arm to measure.

[redacted]

Step 4: Write down the mid-arm measurement in Data Collection Form (DCF).

Step 5: Repeat the mid-arm measurement for the same arm and write down the measurements in the Data Collection Form (DCF).

Step 6: Calculate the average of the two mid-arm size measurements you measured. For calculating the average, you should add the two measurements and then divide their total by 2:

(a + b) / 2 = Average (please write down the average mid-arm size in the Data Collection Form)

For calculation, use a calculator (e.g., located on your smartphone, iPad etc.)

Step 7: Now compare the number you calculated with the cuff size ranges below.

For example, if your average number came out to 18cm, you would select the '***Size 9 green cuff'*** provided in your kit OR if your average number came out to 16cm, you could select either the ***'Size 8 black cuff***' or the ***'Size 9 green cuff'***.

9-13cm Size 7: Darker Black

12-16cm Size 8: Black

15-21 cm Size 9: Green

20-26 cm Size 10: Blue

25-34 cm Size 11: Gray

32-43 cm Size 12: Red

Make sure you select the correct cuff size for accurate blood pressure measurement. If you are using **Size 7 or Size 8** cuffs, please double check the cuff before use as both **Size 7 and Size 8** cuffs are black in color.

Step 8: Mark the appropriate blood pressure cuff size listed in the Data Collection Form (DCF).

Step 9: Place the BP cuff around the arm at the level of the heart. Apply the cuff, allowing room for no more than two fingers. Insert the end of blood pressure connection tube into the cuff as shown below.

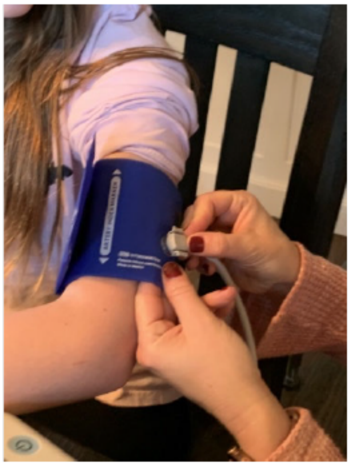

Step 10: Once the cuff is placed, allow the child to sit for **five minutes.** You can have the child watch a familiar video, play with their favorite toy, or watch a sibling to help while measuring the child.

Step 11: Press the start button on the machine and wait for the machine to display the numbers of the screen.

Step 12: During this time, check the child's positioning. Arm and fingers should be relaxed, palm facing up, cuff at heart level, and make sure the cuff is secure. The measurement will take 20 seconds.

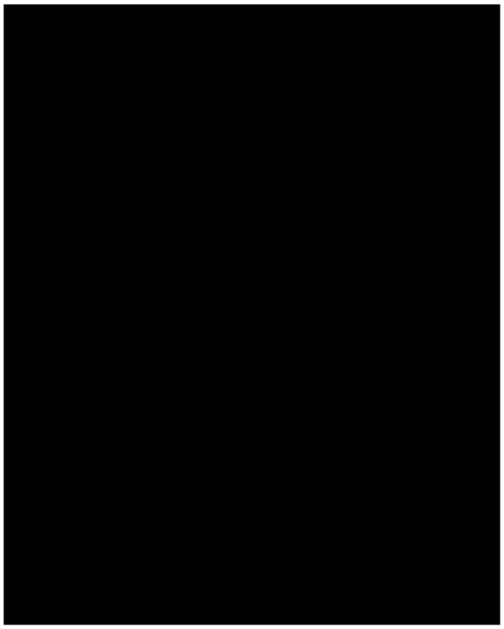

Note: If the child is not sitting still, you will see the sign as shown in the picture below.

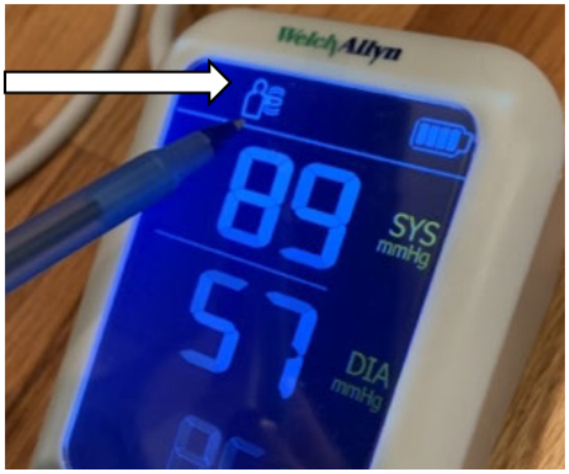

Step 13: Write down the SYS (Systolic BP), the DIA (Diastolic BP), the pulse rate in the instrument in section Data Collection Form (DCF) provided to you. After this step, you can remove the child's cuff.

Step 14: Repeat steps (8-13) two more times in 1-minute intervals between each measurement and write down a total of three SYS (Systolic BP), the DIA (Diastolic BP), the pulse in the instrument in the boxes with the same name next to it on the Date Collection Form (DCF).

Step 15: After the third measurement is noted in the Data Collection Form (DCF), remove the BP cuff from the child's arm and the measurement is complete.
